# Driving the implementation of the National Health Act of Nigeria to improve the health of her population

**DOI:** 10.11604/pamj.2023.45.157.37223

**Published:** 2023-08-11

**Authors:** Olayinka Stephen Ilesanmi, Aanuoluwapo Adeyimika Afolabi, Christianah Toluwalope Adeoya

**Affiliations:** 1 Department of Medicine, College of Medicine, University of Ibadan, Oyo State, Nigeria,; 2 Heller School for Social Policy and Management, Brandeis University Massachusetts, Massachusetts, USA

**Keywords:** Health insurance, health policy, health system, Nigeria

## Abstract

The Nigerian government has previously implemented policies to achieve universal health coverage, however, only few are health-insured. In 2022, the President of the Federal Republic of Nigeria passed the bill for the National Health Insurance Act. As a result of this development and the ensuing target of providing health insurance to all Nigerians by 2030, efforts to combat the high prevalence of poverty caused by out-of-pocket medical expenses while engaging with State Health Insurance Agencies are now more feasible than ever. Health insurance is now required for all Nigerians and legal residents. This article thus aimed to outline strategies to ensure that the National Health Insurance Act contributes positively to the health and well-being of Nigerians.

## Perspectives

The provision of effective health services for the preservation and promotion of life has always been a source of concern for all responsive governments around the world. It's no surprise that one of the Sustainable Development Goals (SDGs) of the United Nations aspires to promote good health and well-being for people of all ages (Sustainable Development Goal 3) [1]. The Nigerian constitution also commendably guarantees everyone the right to sufficient medical and health care. On 15^th^ October, 1997, the National Health Insurance Scheme was inaugurated in Nigeria, and it was approved into law in May 1999 [2]. The National Health Insurance Scheme Act of 1999, the National Health Insurance Act of 2014, and the amended National Health Policy of 2016, are some of the laws and policies that the government have implemented to ensure access to health care for all Nigerians [3,4]. However, on 24^th^ May, 2022, the President of the Federal Republic of Nigeria passed the National Health Insurance Act bill, which was developed to replace the 2014 National Health Act [4]. The National Health Insurance Scheme´s major goal is to provide affordable healthcare to insured people and their dependents, allowing for simple access to healthcare in the pursuit of universal health coverage for all [4]. It is a prepayment system that provides a minimal level of economic security in the event of unfavorable losses due to accidents, sickness, old age, unemployment, and other factors [4]. Some states in Nigeria have developed state health insurance agencies; however, only about 3% of individuals were expected to be covered, owing to the mandatory enlistment of government employees, leaving many civilians uninsured [5]. As a result of the development of the National Health Insurance Act bill and the ensuing target of providing health insurance to all Nigerians by 2030, efforts to combat the high prevalence of poverty caused by out-of-pocket medical expenses while engaging with state health insurance agencies are now more feasible than ever [4]. The new National Health Insurance Act bill was passed to promote, regulate, and integrate health insurance programs in Nigeria, as well as to increase and harness private sector engagement in healthcare delivery [4]. Health insurance is now required for all Nigerians and legal residents under the new law, which also establishes a basic minimum package of health care for all Nigerians across all health insurance schemes functioning in the country [4]. The new Act also creates a Vulnerable Group Fund for children under the age of five, pregnant women, the elderly, individuals with physical and mental disabilities, and the impoverished, thus ensuring equity in healthcare access [4].

Affordable healthcare for the people living in low- and middle-income countries is a recurring developmental challenge. Health financing for the cause of universal health coverage has helped to achieve great strides in the health sector in some developing countries, including Thailand, Mexico, Moldova, Rwanda, and Ghana [6,7]. Thailand attained universal coverage in 2002, following the newly elected government's introduction of the ''30-Baht for All Diseases Policy'' in 2001 [6]. The 30-Baht policy established a universal coverage scheme to cover over 45 million Thais who were not already insured by the civil servant medical benefits package and the social security scheme and only required a 30-Baht (about $1) copayment for each visit [6]. One of the main objectives of Ghana's national health insurance scheme was to improve access to and utilization of pharmaceuticals and other health services, especially among vulnerable populations [8]. Witter and Garshong reported a dramatic increase in the number of outpatient visits shortly after the commencement of the health insurance scheme in 2005 [8]. In Rwanda, the success of the *mutuelles de Santé* program has made the sub-Saharan African nation foremost in the region, having the largest enrollment in health insurance [9]. To reach the desired goal of equity in healthcare access, the implementation of the National Health Insurance Act need not be delayed any further. Prompt action will be beneficial to maximize the benefits present therein and improve the quality of healthcare for the residents of Nigeria. This perspective article therefore aimed to outline strategies to ensure that the National Health Insurance Act contributes positively to the health and well-being of Nigerians, while drawing on experiences from the health insurance system of other countries.

**Lessons from the United Kingdom National Health System:** the National health system is the free, publicly funded healthcare system of the United Kingdom [10]. The World Health Organization (WHO) estimates that government funding accounts for 85% of healthcare costs in the UK, with the private sector funding the remaining 15% [10]. The National Health System differs from many other healthcare systems in that it is not supported by health insurance as is commonly known, but rather by taxes. To aid implementation of the National Health Insurance Act, it is important to draw lessons from the model of the National Health System in England and how it has managed the provision of access to care for all its inhabitants and the regulation of service providers. The UK National health system operates in tandem with several other organizations, the synergistic effect of which is the provision of quality care. Some of these organizations include the Care Quality Commission, which is responsible for monitoring and enforcing safety and quality standards; the National Institute for Health and Care Excellence, which is responsible for setting effective treatment guidelines as well as assessing the cost-effectiveness of new health technologies; and the National Health System Improvement Education England, responsible for planning the National Health System workforce.

**Lessons from the United States of America:** the ''Affordable Care Act'' refers to the comprehensive health care reform law enacted in March 2010 [11]. The goals of the Act are to lower healthcare expenses for households earning between 100% and 400% of the federal poverty threshold, cover all people with incomes below 138 percent of the federal poverty level while expanding the Medicaid program, and encourage cutting-edge medical care delivery strategies intended to reduce overall health care expenditures [11]. It was created with the lofty objectives of enhancing accessibility, affordability, and quality of health care. Since then, the uninsured rate has decreased by 43%, from 16.0% in 2010 to 9.1% in 2015, and there are signs that both financial security and health status are improving [12]. The Act created the concept of accountable care organizations (private insurers), an integrated healthcare delivery system that encourages resource efficiency to cut back on wasteful spending [13].

**Issues requiring urgent attention for the National Health Insurance Authority to have the expected impact in Nigeria**

**Corruption:** according to Transparency International´s report for 2021, Nigeria had a corruption perception index score of 24 out of 100, the lowest it has had since 2012 [14,15]. This ranks Nigeria as one of the most corrupt countries globally [15]. This ranking takes into consideration, particularly, the level of corruption in the public sector in various countries across the world. Unfortunately, Nigeria´s healthcare sector is not exempt from the wild arms of corruption sweeping across the length and breadth of existing systems, agencies, and schemes in the country. Misappropriation and misuse of resources are two of the greatest challenges affecting the potential success of Nigeria´s health insurance, resulting in significant losses. Insurance fraud in the health sector manifests in fraudulent claims, inflation, or exaggeration of claims, and dishonest actions to obtain more than one's legal entitlement, amongst others. Corruption and fraud have been previously reported in the National Health Interview Survey (NHIS) and the Nigerian healthcare system at large [15,16].

**Fragmentation of health insurance:** as stated in the National Health Insurance Act, the act will run a scheme that exclusively provides health insurance for civil servants in the federal service. Thus, it seems biased if this Act, which is tasked with the responsibility of providing oversight for other schemes, proceeds to run a fragmented pool while implementing health insurance for federal employees across the nation [4]. While federal employees' paychecks are organized and paid centrally, it is obvious how a separate program may be more practical for the simplicity of premium contributions. As a result, deduction at the source is simpler [17]. However, fragmentation of health insurance should be discouraged. To address this, a deduction of federal employees' health insurance at the source and payment to the appropriate state health insurance programs will be beneficial. This will enable the National Health Insurance Act to effectively regulate health insurance in Nigeria by consolidating the State Health Insurance pools.

**Poorly structured primary, secondary, and tertiary health care:** the National Health Insurance Act is an opportunity to make things right in the structure of health care in Nigeria [4]. In the current structure, it seems that primary, secondary, and tertiary care are just nomenclatures. Patients that are meant to be managed at primary health care centres are treated at tertiary hospitals, and those meant to be referred from tertiary back to primary health care centres are not [18]. The three tiers of government in Nigeria are simultaneously responsible for the provision of healthcare. The confusion in the structure can be addressed through the National Health Insurance Act. Beginning with the most fundamental level of care, primary health care centres in Nigeria are typically found to have limited and outdated facilities. They only serve to identify and treat mild ailments, and as such, are modestly sized. The typical primary health care settings are not available round the clock every day and care are offered in a public or communal context [19]. With secondary health care facilities, the patients should receive specialized medical care [20,21]. In addition to treating specific disorders, they offer more sophisticated care than primary healthcare. The secondary facilities are affected with incessant strike actions and poor resources in Nigeria. As a result, patients are directly referred to the tertiary health facilities from primary health centres. It seems the secondary health care has lost its importance. Tertiary health care is concerned with the provision of specialist or broad treatment for illnesses. Although they can offer patients consultation services, they do address general cases. Tertiary facilities are overburdened with too frequent cases due to the weakness of the primary and secondary health systems.

**Inadequate funds and human resources for health:** the World Bank predicted that by the end of 2018, worldwide remittances will have increased by 10.3%, totaling 689 billion US dollars, with about 528 billion of those dollars flowing to developing nations [22]. Remittances have grown to be one of the most important sources of capital inflow in Africa, contributing to the economic development and standard of living of the host country. Nigeria is the top recipient country of remittances in sub-Saharan Africa [22]. Nigeria is a major exporter of both unskilled and, more crucially, trained, and professional labor, particularly to industrialized nations in the healthcare industry [22]. Amidst the rising state of insecurity in Nigeria and other unfavorable economic and work conditions for these health professionals, there is no doubt an increasingly alarming rate of emigration of healthcare professionals to what is deemed ‘greener pastures´ has been seen on the part of the government to mitigate it [23]. More than 90% of health centers and general hospitals have no healthcare professionals, especially medical doctors [23]. As of 2018, a poll run by NOIPolls showed that about 88% of doctors are considering work opportunities outside the country, with 87% of the respondents in that survey believing that the Nigerian government is largely unconcerned with the move and the ensuing implications [23]. Push factors for the mass exodus of Nigerian healthcare workers include poor payment packages, especially for early and middle-career healthcare professionals, irregular payment patterns, and poor working conditions, while pull factors such as excellent working conditions and regular payment patterns continually attract skilled Nigerian healthcare workers to “greener pastures” [24]. There is a need to address the issue of low manpower in the health workforce in Nigeria.

**Political interference in executing public health programs:** politics is frequently described as the subway´s third rail in the promotion of public health and the execution of health interventions. Many giant strides in global health have been achieved because of political engagement, some of which include a reduction in exposure to second-hand smoking, removal of lead from gasoline and the resultant fall in blood lead levels in children, and public mandates for the use of seat belts and other traffic standards [25,26]. Political interference in public health programs still exists, thus limiting the promising successes of those programs. As elucidated by Kelsall *et al*. in their research, politics should be synchronized with current trends in science to construct consistent programs and policies to achieve universal health coverage [27]. Many low and middle-income countries, including Nigeria, suffer from improper prioritization and lack of strategic plans by political actors, and these deficits cause impediments to the sustainability of healthcare programs and attainment of universal health coverage. Rather than siting health facilities to score political points, policymakers and constituted authorities should adopt proactive measures to conduct health needs assessments for the people they represent and set up needs-specific infrastructure. Governance has been identified by the World Health Organization to be positively associated with universal health coverage. Hence, the transparency, prompt delivery, and efficiency of political actors and other stakeholders need to be significantly improved upon to facilitate health service and national health insurance coverage as suggested by the National Health Insurance Act.

## Conclusion

The National Health Insurance Act is equipped with a variety of new, extended functions that include its empowerment as an insurer of registered private health companies; a regulator of health maintenance organizations and other third-party administrators; and an investor of health funds to provide financial support during emergencies. However, the functional capacity of the National Health Insurance Act as an implementer is a core component that did not record significant modifications from the NHIS Act of 1999. While a change in nomenclature from National Health Insurance Scheme to the National Health Insurance Act is well acknowledged, modifications in implementation style and functional capacity are needed to achieve the potential success promised by the Act. Good governance, political stability, demographic considerations, and stakeholder engagement are requirements for achieving universal health coverage and good national health insurance coverage easily. Since universal health coverage is a goal, those in positions of authority, such as legislators, policymakers, and program implementers, must take ownership of it. Extensive information and data management system should be built by the National Health Insurance Act in partnership with other information management organizations in Nigeria and across all states for effective combination and regulation of health insurance schemes by the National Health Insurance Act. A phase-by-phase implementation of the National Health Insurance Act is needed to maximize its benefits. A payment method that consciously sets incentives for quality improvement and clearly defined service packages as key features for strategic purchasing should also be developed. Good governance, political stability, demographic considerations, and stakeholder engagement are requirements for achieving universal health coverage and good national health insurance coverage easily. Since universal health coverage is a goal, those in positions of authority, such as legislators, policymakers, and program implementers, must drive it. An extensive information and data management system should be built by the National health insurance act in partnership with other information management organizations in Nigeria and across all states for effective combination and regulation of health insurance schemes by the National health insurance act. A phase-by-phase implementation of the National health insurance act is needed to maximize its benefits. A payment method that consciously sets incentives for quality improvement and clearly defined service packages as key features for strategic purchasing should also be developed.
